# Environmental health in Australia: overlooked and underrated

**DOI:** 10.1093/pubmed/fdy156

**Published:** 2018-10-05

**Authors:** H Whiley, E Willis, J Smith, K Ross

**Affiliations:** 1 Environmental Health, College of Science and Engineering, Flinders University, Adelaide; 2 Health Sciences, College of Nursing and Health Sciences, Flinders University, Adelaide

**Keywords:** climate change, planning, public health, workforce

## Abstract

Improvements in environmental health have had the most significant impact on health status. In Australia, life expectancy has significantly increased through provision of vaccination, safe food and drinking water, appropriate sewage disposal and other environmental health measures. Yet the profession that is instrumental in delivering environmental health services at the local community level is overlooked. Rarely featuring in mainstream media, the successes of Environmental Health Officers (EHOs) are invisible to the general public. As a consequence, students entering university are unaware of the profession and its significant role in society. This has resulted in there being too few EHOs to meet the current regulatory requirements, much less deal with the emerging environmental health issues arising as a consequence of changing global conditions including climate change. To futureproof Australian society and public health this workforce issue, and the associated oversight of environmental health must be addressed now.

## Introduction

Environmental health is the discipline that has had the greatest impact on human health. The World Health Organization estimates that 15 of the extra 20 years of life that we now enjoy compared with a century ago can be attributed to environmental health interventions.^1^ It is also estimated that 26% of deaths among children under five can be attributed to modifiable environmental risk factors such as air, water and soil pollution chemical exposure, climate change and UV radiation.^2^ Furthermore, it has been estimated that by 2030 climate change is expected to cause an estimated 250 000 additional deaths per year, from issues requiring environmental health solutions including malnutrition, malaria, diarrhoea and heat stress. Despite being one of the most essential professions for protecting human health, environmental health is under-recognized, overlooked and misunderstood.^3,4^ This article explores the reasons for this oversight and appeals for greater recognition, primarily to attract practitioners to the profession in order to deal with emerging issues that will require future environmental health graduates.

## History of environmental health

There are many definitions of public health, and the importance of environmental health within these depends on what is in vogue at any particular time in history. Environmental health is based on the perspective that while health and illness impact on individuals, the origins of disease and good health are socio-political^5–7^ so the focus must be on populations, legislation, monitoring, investigation and surveillance.^8,9^ This means that much of the public health work is outside of the traditional healthcare system and beyond the scope of most doctors, nurses and allied health professionals. One of the difficulties for environmental health is that the golden age of public health advances is assumed to have been the late 19th and early 20th centuries when the major disease-causing risk factors associated with poor housing and sanitation and contaminated water supplies were tackled. For example, research by McKeown^10^ demonstrated that reductions in the rates of population morbidity and mortality in Britain occurred well before the invention of antibiotics, or mass immunization campaigns for diseases such as tuberculosis. These reductions were a direct result of programs in sanitation, clean water, safe working conditions and adequate housing. One of the key critiques of McKeown’s thesis comes from the work of Szreter.^11^ He makes the point that these health improving interventions did not occur in a vacuum, but rather were the result of the work of local governments, political battles and ideological struggles to ensure legislation and protections were put in place to maintain healthy environments across cities and regions.^12^ This is the space occupied by environmental health.

Developments within the public health movement in the last 20 years have taken a number of contradictory turns that have also impacted on how environmental health is positioned within the broader health arena and hence on the status accorded to the profession. One direction has been to shift the focus of public health away from political, legislative action and surveillance towards seeing it as an individual responsibility.^13,14^ This approach foregrounds lifestyle and encourages behavioural approaches to public health problems such as obesity, smoking, drug misuse and sexual health.^15–18^ However, as identified in the health impact pyramid by Stewart and Hursthouse,^19^ public health interventions which have the greatest impact on health at a population level are those that address environmental factors. Another movement has been the swing back towards social factors that occurred firstly with the Health For All movement,^20^ and more latterly with the World Health Organization’s Social Determinants of Health agenda.^21^ This is known as the new public health movement.^12^ Proponents of new public health argue that the agenda has broadened out beyond concerns for the physical infrastructures, or issues of clean food, and legislative controls, to champion social support for population groups. Social supports situate the problems of illness and disease in inequality arising from socio-economic status, gender, race, geography, ethnicity, access to transport, education and affordable housing. While there is no denying the link between health and these social factors of inequality, there is an assumption that the protective legislation and infrastructure is in place. What is not readily understood is that it is these very populations who become the victims of any failure in environmental health protections.

A third factor impacting negatively on environmental public health work has been the rise of neo-liberalism.^22,23^ The majority of Environmental Health Officers (EHOs) in Australia are employed by local councils.^24,25^ This third tier of government has been subject to major managerial reforms in the interests of increased productivity and efficiency since the late 1980s.^26,27^ This has seen a rationalization of EHO positions across the sector while the scope of their role has increased. It has also meant that budget pressures have restricted the extent to which EHOs have time to become involved in important but non-regulatory responsibilities, such as planning (Psarras, pers comm. 2018). In addition to these seemingly contradictory approaches there is competition for resources required to deliver environmental health services and competing policy interests within environmental health and specific service areas, e.g. food safety, which impact on practice and practice standards (Smith, Ross, Whiley 2016).

There is also the propensity of the environmental health profession to underrate and undersell themselves. This is reflected by EHOs feeling undervalued by employers (state and local government) and the community,^28^ resulting in poor job satisfaction and retention rates.^29^ As such, there is a need for more professional development supporting leadership and advocacy of the EHO role.^25^ The broad scope of the EHO role (Table 1) also means that the environmental health profession struggles to elucidate a single unifying characteristic. The role of the EHO is to identify and characterize risks to human health and then propose an approach to resolving the issue to prevent people from becoming ill or injured.

**Table 1:** Roles and responsibilities of Australian environmental health officers (EHOs) adapted from EnHealth^29^

*EHO roles/responsibilities as an Authorized Officer (AO)*^a^
Register, license or approve applications for environment related activities that can affect public health
Assess health hazards and risks to confirm compliance with legislation and any related registration or licensing condition
Undertake investigations
Determine and direct or implement remedial action
Emergency management
Enforce legal requirements and prosecute legal cases
*EHO roles/responsibilities applying to multiple functional areas*
Risk management
Communication, promotion and education
Health promotion and education
Emergency and incident management
Climate change
Planning: environmental health
Contract and project management
Health and safety
Indigenous health
*EHO roles/responsibilities applying to specific functional areas*
Food safety
Notifiable conditions/infection control
Water management
Environmental protection
Planning: development
Structures/buildings, accommodation
Tobacco
Control of drugs and poisons

^a^State or local government authorize EHOs to administer the provisions of the individual acts of parliament.

## The invisible profession

**Fig. 1:**
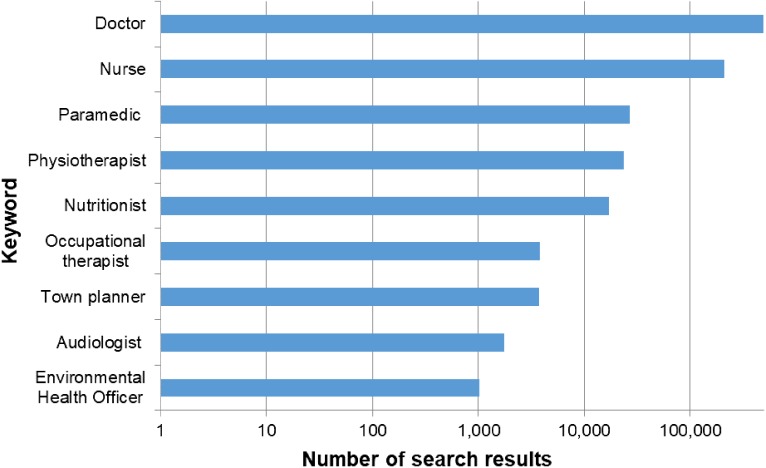
The number of media articles (note log10 scale) found from 2007 to 2017 on the Australia and New Zealand Newsstream (ProQuest) database using the professions as keywords for the searches.

The invisibility of the environmental health profession may be a consequence of EHOs being altogether too good at their jobs, resulting in consistently achieving success in the preventative nature of their work. Unlike a medical practitioner where the benefits of their intervention are often immediately apparent to the individual, the benefits of an EHO doing his or her job successfully are likely to result in an issue never becoming a problem. This under-recognition of the environmental health profession can be illustrated by their lack of appearance in mainstream media. A search was conducted using Australia & New Zealand Newsstream (ProQuest) focusing on different health professions as keywords and the number of media articles containing that profession from 2007 to 2017 is shown in Fig. 1. ‘Doctor’ features the most heavily in the media returning 497 502 articles and Environmental Health Officer received the least number of search results with 1025, even fewer than smaller health professions such as audiologist (1754) or occupational therapist (3865) and other local government professions such as town planner (3756).

This lack of recognition from the media is also echoed by the Australian Federal Government, illustrated by the Australian Taxation Office’s absence of a job code for Environment Health Officer. This is despite having job codes for over 55 different types of nurses. Although there is a code for health inspector (251 311),^30^ this is a term that has not been in use in Australia for over 25 years and may point to issues around role ambiguity. This change from ‘health inspector’ to ‘environmental health officer’ was an international change that occurred as the profession moved to a minimum degree level qualification and associated increased responsibilities.

**Fig. 2:**
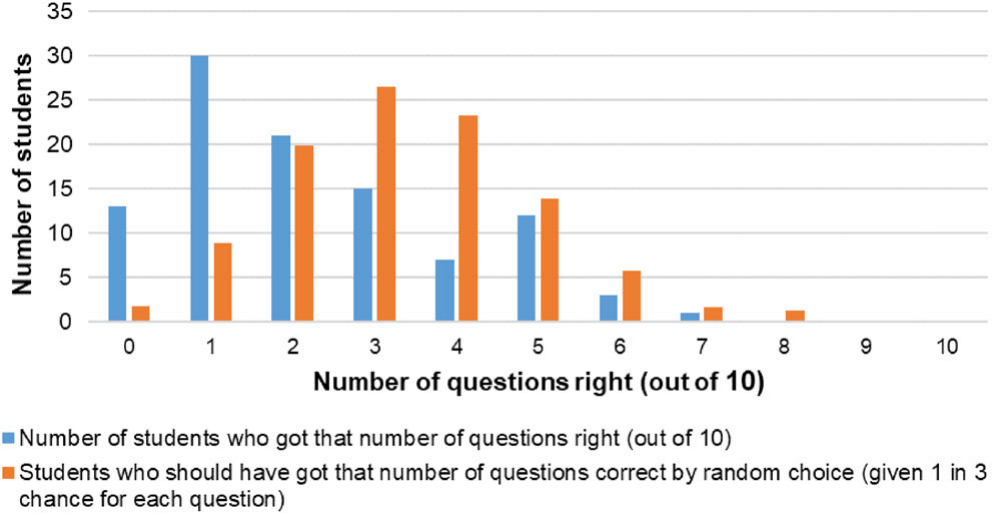
Number of who got answers correct (out of 10 question) compared with the probably of student getting that number of questions correct based on random chance (given one in three chance for each question).

A consequence of the profession’s lack of visibility is that too few students undertake environmental health degrees, resulting in a shortage of EHOs.^24^ This is illustrated by a survey completed by 103 first year students at a South Australian University (ethics approval provided by the Flinders social and behavioural research ethics committee—project no. 7859). The students were enroled in a first year science topic not related to environmental health. They were presented with a series of short activities (Appendix 1) and were asked to respond yes/no/unsure if they thought the activities was the responsibility of a local government EHO. The results (Fig. 2) show that the students got the questions wrong more times than if they have just answered randomly.

## The wider impacts of under-recognition

A consequence of the under-recognition of the environmental health profession is that responses to public health issues are often dominated by a clinical perspective with the impact of the environmental and preventative measures overlooked. A good example of this is the management of the cholera outbreak in Haiti in 2010. The cholera was brought into the country by Nepalese peacekeepers with asymptomatic infection; however, the catastrophic outbreak was a consequence of improper sewage treatment resulting in contamination of waterways. The clinical-focused response to this disaster was to screen all peacekeepers ‘had the UN screened its Nepalese peacekeepers, for a cost of only about $2000, this crisis could have been averted’.^31^ Paradoxically, the environmental health response was that wastewater management is essential for protecting human health and faecal matter should never contaminate drinking water regardless of whether it contains cholera or not. ‘This epidemic reminds us how critical the management of water and sewage is to prevent cholera spread. To avoid actual contamination or suspicion happening again, it will be important to rigorously ensure that the sewage of military camps is handled properly’.^32^

An example in Australia is strongyloidiasis, which remains recalcitrantly high in Indigenous Australian communities.^33^*Strongyloides* infection should serve to highlight failing wastewater disposal systems, inadequate rubbish collection and disposal, chronic overcrowding in housing and inadequate access to appropriate veterinary care. However, these environmental health aspects of healthcare are often overlooked in favour of clinical treatment with ivermectin.^34^ This success of ivermectin to treat strongyloidiasis has served to diminish advocacy for better housing hardware and services. This is concerning because ivermectin treatment does not prevent reinfection and the ongoing struggle to control *Strongyloides* infection demonstrates that clinical intervention alone is inadequate.^35,36^

## Emerging risks

The greatest emerging threat to global public health is climate change.^37^ Climate induced changes in the severity and frequency of natural disasters such as floods, storms, fires, heat waters and seasonality of infectious disease outbreaks will require emerging and evolving environmental health solutions.^38^ From 2000 to 2015 the frequency of weather related disasters increased by almost 50%, and this number is expected to rise.^39^ This in turn will impact on food and water security and have the social and economic consequences of mass displacement of populations.^40,41^

EHOs need to be resourced and supported to be involved in emergency management and public health planning to ensure the impact of the changing environment on human health is considered and planned for at the community level. This is essential as Australia develops climate policy and legislation to protect the country and its future.

## Moving forward

Clearly, EHOs need to be recognized and valued as a vital part of the professional public health workforce. Indeed, in Australia they are the only professionals providing health protection services to the community at the local level.^3,24,28^ Although these health protection services are required of local government by state statutes,^42^ nonetheless there are a range of skills required to administer the legislative requirements effectively and efficiently through the development of educational, informational and enforcement strategies. As regulators, EHOs are focussed on how best to achieve changes in behaviour both in industry and the broader community and achieve legislative objectives, e.g. safe food production, which is in the public interest. Efforts to change behaviours need to be considered and managed skilfully to meet not just legislative objectives but also local community expectations and demands, and the organizational values and strategic goals of the employing council.

The challenge for EHOs is to continue along the professionalization pathway which was initiated by the introduction of qualifying university degree programs in Australia over 30 years ago.^29^ This pathway demands that legislation is not the driver of, or the *raison d’etre* for, the EHO profession but the development of practice and professional standards becomes the focus. The development of practice requires, as with other professions, the creation of ethical and best practice standards, peer processes for improving professional practice, and a certification scheme for EHO practice. Professional certification, or registration, might address, at least in part, the overlooked and underrated profession of environmental health officer. There is also a need for the development of a national strategy to raise the profile of environmental health and increase the value of this workforce especially to Local Government Senior Management and CEOs. This links back to a professional certification scheme supporting the development of leadership and advocacy skills.^28^

## Conclusion

Environmental health as a profession has historically been overlooked for its positive impact on health status. It is a profession that is needed as much now as it ever has been if we are to maintain the longevity and high health standards we now expect. It is imperative that we find methods to enhance the visibility of the profession and thus, attract and maintain the next generation of EHOs.

## Supplementary Material

fdy156_Appendix_1
