# Supplementary material for: Environmental health in Australia: overlooked and underrated
Source: J Public Health (Oxf). 2018 Oct 5;41(3):470–5. doi: 10.1093/pubmed/fdy156 (PMC6785703; doi:10.1093/pubmed/fdy156)
Supplement: fdy156_Appendix_1 [file fdy156_appendix_1.pdf]

Appendix 1: Survey questions presented to first year University science students

Which of the following issues are the responsibility of environmental health officers…..

Monitoring of logging permits to prevent deforestation

☐ Yes ☐ No

Clean-up of clandestine laboratories

☐ Yes ☐ No

Preventing the spread of disease through koala populations

☐ Yes ☐ No

Ensuring that food is stored at the correct temperatures in restaurants

☐ Yes ☐ No

Ensuring tattooing equipment is sterilised properly

☐ Yes ☐ No

Monitoring of wheat crops to prevent the spread of crop disease

☐ Yes ☐ No

Management of on-site waste water applications

☐ Yes ☐ No

Management of initiatives preventing the pollution of the oceans

☐ Yes ☐ No

Ensuring recycling schemes operate effectively

☐ Yes ☐ No

Mediation of hoarding cases

☐ Yes ☐ No

Investigation into legionnaire's disease outbreaks in the community

☐ Yes ☐ No
